# Penitentiary Patients

**Published:** 1890-03-29

**Authors:** 


### Penitentiary Patients.

The Church Penitentiary Association recently held a special conference at the Church House, Westminster. A large number of ladies engaged in rescue work, the chaplains of penitentiaries, and some lay gentlemen, composed an assembly which completely filled a large hall. In the absence of the Bishop of Marlborough, the chair was taken by Mr. J. C. Thynne, Hon. Treasurer of the Westminster Refuge.

Sister Zilla (East Grinstead), for many years head of Newport Market Refuge, contributed a paper in which suggestions were offered for the greater efficiency of penitentiary work. There was a great want of convalescent homes at present. These she wished to be carefully distinguished from hospitals, where girls went in extreme and perhaps fatal illness. She specially referred to convalescent homes, where those who were free from disease, and only weak and ailing, might be sent, to be built up either after they had left the hospital or a penitentiary. In such homes discipline would have to be relaxed to a certain extent, and strengthening food, fresh air, with no hard work, would be necessities. Servants who had regained their character, but whose health was broken down, and who needed temporary rest, might be received on payment of a small sum, and go back to their work with renewed health and strength. Discipline, of course, there must be, but mixed with more than ordinary gentleness and kindness. In this stage of convalescence the patients were specially alive to kindly influences, and the opportunity might be utilized with the utmost advantage for raising their fallen sisters to higher and better things. Along with bodily strength, they might gain in this interval even something better than bodily strength to help them when they had again to face a life of temptation and care. Many a girl, safe while busy with work, fell again in a time of idleness, brought about perhaps by illness, and it would be of untold benefit that at such seasons there would be the alternative of going to a home to regain her strength, instead of falling back into the old life which she had left. There were many convalescent homes, but only one (so far as she knew) for the class of women now spoken of. Many who knew what it was to feel sick and ailing, and who had recovered strength, might out of their abundance, as a thankoffering, help in procuring these convalescent homes. There were at present two institutions (St. Agnes' Hospital, for the very sick, and the Home of Rest at Balham for convalescents) which, if well supported and enabled to widen their borders, might greatly meet the need which existed. There were so many new works undertaken that she had no desire to increase the number, and so suggested an enlargement of present places. S. Mary's Sisters, at New York, had a penitentiary where all were received, the sick and weakly ones being sent to a sanatorium on a neighbouring island.

The Rev. H. Ampse, chaplain of St. Mary's Home, Highgate, said he believed the small home at Balham was not always full. He thought, in any enlargement of present plans, it would be well to have branch homes at the seaside. Girls who faithfully did the work to which they had returned and kept their own council, were received into the ordinary homes; but for those who were in penitentiaries, or taken off the street, such convalescent homes as were suggested would be of the greatest service. A place like St. Agnes' Hospital was most valuable in this work, for sending a girl to the lock wards or general hospitals was something like losing the girl; she mixed up with girls who had come from an evil life and meant to return to it again, and the result was deplorable. They wanted more places like St. Agnes' Hospital, where their patients would be thoroughly looked after by the sisters.

The Chairman said that the Home at Balham was originally intended for the reception of girls from the general hospitals, girls who would not be received if their character was known by the ordinary convalescent homes. That home might be enlarged. St. Agnes' Hospital was even more, perhaps, the kind of place required. Girls were kept there for many years after their recovery. Like the home at Balham, it had not been as well supported as it should be, because the existence of these places was not sufficiently known.

General 'Newmenheere spoke of St. Mary Magdalene's Home, Paddington, where girls were retained for a considerable time with the best results. He had no confidence in "short time penitentiaries," as they were called. The religious and refining influences of good surroundings were necessary in this work, and required time to prove a lasting benefit.

The Rev. J. Walker, Chaplain of the Magdalene Hospital, Streatham, said they wanted more hospitals where these girls would be taken either from a refuge or from a home. They were sometimes able to get patients into St. Thomas's Hospital and to convalescent homes from that place, but the hospital people did not profess to receive such cases; it was a favour. There ought to be a hospital to which cases from any part of England could be sent; and from thence to convalescent homes, and these homes should be at the seaside. People should know that the lock hospital system had proved a failure.

The Chairman thought that small homes in different places, like the one at Balham, would be better than one large central home. As to the short time system, they must remember that the disposition of no two women was exactly alike. At Westminster Refuge they found it hard to get girls to go to a home; sometimes they would go for a year, sometimes for six months, but any really long period would probably be resented. Even in a short time, if well managed, the girls received benefit from their stay in a refuge or home. He concluded it was the opinion of the conference—and no resolution would be submitted—that they ought to advance on the lines followed by the promoters of St. Agnes' Hospital and of the Balham Home of Rest.

An address on the more directly spiritual character of penitentiary work was then given by the Rev. P. F. Davidson, chaplain of St. James' Home, Fulham.

